# Toxicity of diclofenac sodium salt in Yucatan minipigs *(Sus scrofa)* following 4 weeks of daily intramuscular administration

**DOI:** 10.1016/j.toxrep.2021.02.022

**Published:** 2021-02-26

**Authors:** Hyung-Sun Kim, Goo-Hwa Kang, Mi-Jin Yang, Hee-Jeong Ahn, Su-Cheol Han, Jeong Ho Hwang

**Affiliations:** a Animal Model Research Group, Jeonbuk Branch Institute, Korea Institute of Toxicology, Jeongup, Jeonbuk, 56212, Republic of Korea; b Jeonbuk Pathology Research Group, Jeonbuk Branch Institute, Korea Institute of Toxicology, Jeongup, Jeonbuk, 56212, Republic of Korea; c Jeonbuk Quality Assurance Unit, Jeonbuk Branch Institute, Korea Institute of Toxicology, Jeongup, Jeonbuk, 56212, Republic of Korea; d Jeonbuk Branch Institute, Korea Institute of Toxicology, Jeongup, Jeonbuk, 56212, Republic of Korea

**Keywords:** AAALAC, association for assessment and accreditation of laboratory animal care, ALT, alanine aminotransferase, AST, aspartate aminotransferase, AP, Alkaline phosphatase, AUC, area under the concentration-time curve, aVF, augmented vector foot, aVL, augmented vector left, aVR, augmented vector right, BW, body weight, BUN, blood urea nitrogen, CAS, chemical abstracts service, CL, clearance, C_max_, maximum plasma concentration, COX, cyclooxygenase, CREA, creatine, CRO, contract research organization, CV, coefficients of variation, DSS, diclofenac sodium salt, ECG, Electrocardiogram, GI, gastrointestinal, GLP, good laboratory practice, H&E, hematoxylin and eosin, HED, human effective dose, HR, heart rate, IACUC, institutional animal care and use committee, NDA, new drug application, NOAEL, No-observed-adverse-effect levels, NSAIDs, nonsteroidal anti-inflammatory drugs, RBC, red blood cell, SD, standard deviation, T_max_, time to maximum plasma concentration, TP, total protein, TK, toxicokinetics, WBC, white blood cell, Diclofenac sodium salt, Minipig, No-observed-adverse-effect level, 4-Week repeated toxicity

## Abstract

Diclofenac sodium salt (DSS) is a widely used nonsteroidal anti-inflammatory drug. The present study was performed under good laboratory practice (GLP) regulations to investigate the toxicity of DSS after 4 weeks of repeated intramuscular administration at doses of 0, 2, 10, or 20 mg/kg/day in 32 minipigs and to evaluate the DSS effect following a 2-week recovery period. Dose-related clinical signs and alterations of hematological or clinical chemistry parameters, organ weight, and macroscopic as well as histopathological findings in hepatic, renal, gastrointestinal, skin and injection sites were observed in both sexes’ animals of the 10 or 20 mg/kg/day group. With the exception of the skin-related findings, most symptoms showed a tendency to resolve after the 2-week recovery period. The systemic exposure (AUC_last_) of DSS in plasma showed similar pattern to the increase rate of the dose and similar values between males and females except for the female 20 mg/kg dose group (56 %) on Day1. The systemic exposure showed a decreasing trend in the 10 or 20 mg/kg group after 4-week of repeated administration compared to Day1. The no-observed-adverse-effect level of DSS in this study was considered to be 2 mg/kg/day in both male and female minipigs.

## Highlights

• Four-week repeated-dose toxicity of intramuscular DSS was studied in minipigs.
• DSS administration at ≥10 mg/kg/day causes toxicity and injection-site reaction.
• The NOAEL of DSS after 4-week administration was 2 mg/kg/day in minipigs.

## 1 Introduction

Diclofenac is a nonsteroidal anti-inflammatory drug (NSAID) for the treatment of pain, arthritis, and gout not only in humans but also in animals. It inhibits the synthesis of prostaglandins from arachidonic acid by inhibiting the activity of cyclo-oxygenase 2 [[1], [2], [3], [4], [5], [6]]. However, various adverse effects of diclofenac sodium salt (DSS) have been reported, including gastrointestinal (GI) tract-related symptoms (e.g., nausea, abdominal distress, gastritis, and vomiting), skin-related symptoms (e.g., urticaria and itching and redness of the skin), renal injury, liver injury, and other nonspecific symptoms (general weakness) [[7], [8], [9], [10]].

Nonclinical studies in various species revealed that most adverse DSS-related side effects generally occurred in the renal, hepatic, and gastrointestinal systems. For example, hepatobiliary, nephro-, and GI toxicities were observed following 28 days of repeated oral administration in mice (9.5 mg/kg/day) [11]. In rats, the oral or intramuscular administration of DSS for 2–24 weeks (0.25−40 mg/kg/day) resulted in GI toxicity, lymph node hypertrophy and hyperplasia, and anemia as well as changes in hepatic or renal function parameters (aspartate aminotransferase [AST], alanine aminotransferase [ALT], Alkaline phosphatase [AP], or blood urea nitrogen [BUN]) [1,[12], [13], [14]]. Hematological effects of DSS in goats or chickens have also been reported [2,15]. In dogs, oral dosing for 30–90 days at 0.5−2.5 mg/kg/day resulted in GI toxicity, anemia, extramedullary hematopoiesis, and lymphadenitis [14]. In rhesus monkeys, oral dosing for 30 days to 6 months at 1−500 mg/kg/day resulted in mortality (>15 mg/kg/day), GI toxicity, and anemia [14]. These nonclinical data suggest that some toxicological responses observed in humans are also observed in animals. However, symptoms such as skin-related problems have not been observed in animals except in minipigs.

In minipigs (Yucatan), DSS administered dermally for 30 days to 6 months resulted in GI toxicity, renal and hepatic toxicity, and skin reactions, such as erythema at application sites [14]. Because no-observed-adverse-effect levels (NOAELs) and toxicokinetic values vary according to the route of administration [16,17], this study was performed to present the detailed toxicity and system exposure results of DSS after 28 days of intramuscular administration in minipigs.

## 2 Material and methods

### 2.1 Guidelines for experimental study

The study was designed to administer repeat-dose DSS intramuscularly in 32 minipigs (Yucatan) to investigate toxicity during a 4-week period. The study was conducted at the contract research organization (CRO) in compliance with Good Laboratory Practice (GLP) regulations; the Ministry of Food and Drug Safety Notification No. 2017-71 Test Guidelines for Safety Evaluation of Drugs; and Annex 2 Repeated Dose Toxicity Study and ICH Harmonized Tripartite Guidelines M3 (R2) Guidance on Nonclinical Safety Studies for the Conduct of Human Clinical Trials and Marketing Authorization for Pharmaceuticals.

### 2.2 DSS

DSS (product No. D6899, CAS No. 15,307-79-6, Sigma-Aldrich, Saint Louis, Missouri, USA) and sterilized water (Lot no. 17S2 F21, Dai Han Pharm, Korea) were used as the test substance and vehicle control, respectively. DSS was formulated by suspending it in vehicle to prepare a stock solution (50 mg/mL). This formulation was prepared once a week and stored at room temperature in the dark. Analyses of formulation stability, homogeneity, and concentration were conducted.

### 2.3 Animals

All study procedures and animal care were performed in accordance with the Association for Assessment and Accreditation of Laboratory Animal Care International guidelines and approved by the Institutional Animal Care and Use Committee of the Korea Institute of Toxicology (KIT-1903−0079). The animals used in this study were 3–5-month-old Yucatan Minipigs (*Sus scrofa*) weighing 10–15 kg. Specific pathogen-free minipigs and minipig diets, sterilized by gamma irradiation, were procured from Optipharm, Co., Ltd. (Korea). The animal room was maintained at the following conditions: temperature, 20–26 °C; relative humidity, 30–70 %; ventilation, 10–20 times/h; and light cycle of approximately 12 h at 300–700 lx.

### 2.4 Experimental design

All animals were randomized and assigned to study groups using the Pristima system (Version 7.4. Xybion Corporation, Lawrenceville, New Jersey, USA) based on body weight stratification after 7 days of acclimation. To obtain data before DSS administration, a 7-day pretreatment was set, followed by an administration period for 4 weeks and a recovery period for 2 weeks. A total of 24 male and female minipigs were assigned to a vehicle control group (three animals/sex/group) and three treatment groups (three animals/sex/group), namely low-, middle-, and high-dose groups that received 2, 10, and 20 mg/kg/day of DSS, respectively. The reversibility of any observed toxicities was assessed in a subset of eight additional minipigs (two animals/sex) in the vehicle control and 20 mg/kg/day dose groups, which were assigned to a 2-week recovery period. Doses were administered at a volume of 0.4 mL/kg based on the most recently measured body weight. DSS was administered intramuscularly to the cervical muscle behind the ear once a day for 28 days.

### 2.5 Observation

#### 2.5.1 Clinical signs

Clinical signs, including mortality, moribund state, general appearance, and behavioral changes, were observed and recorded once a day during the entire study period and twice a day during the administration period.

#### 2.5.2 Body weight and food consumption

Body weight and food consumption of all animals were measured weekly. After separating the animals by installing partitions in the cages, feed was provided individually, and the partitions were removed after consumption. Considering the characteristics of the minipigs, the dropped feed on the floor of the cage was recorded as the feed amount remaining after consumption. However, feed falling on the floor outside the cage was not considered to be residual because it could not be accessed it for consumption.

#### 2.5.3 Ophthalmological and electrocardiography examinations

All live animals were first sedated with ketamine (11−12 mg/kg) and xylazine (2−3 mg/kg) prior to ophthalmological and electrocardiographic examination during the pretreatment period, treatment period (day 24), and recovery period (day 10 or 11). Ophthalmological examinations were conducted by a veterinary ophthalmologist using a slit lamp (XL-1, Ohira Co., Ltd., Japan) and a binocular indirect ophthalmoscope (Vantage Plus Digital, Keeler Ltd., England) after the animals were administered one or two drop of a mydriatic agent (Mydriacyl oph soln 1%, Alcon, Geneva, Swiss) to both eyes.

Changes in the electrocardiograms of the anesthetized animals were recorded using an electrocardiograph (Cardio XP, Bionet Co., Ltd., Korea) by placing limb leads I, II, III and the augmented leads aVR, aVL, and aVF. Cardiac electrocardiogram intervals (QT, QTc, PR, and QRS) and heart rate were measured and analyzed by a veterinarian. QTc was derived at each time point using Bazett’s formula [18,19].

### 2.6 Clinical pathology

#### 2.6.1 Hematology and clinical chemistry

Blood samples for clinical pathology were obtained on the following days: before administration and on day 29 for all animals or day 43 for recovery animals. All animals were fasted for approximately 16 h prior to blood collection, but drinking water was provided *ad libitum*. Blood was collected from the jugular vein and placed into EDTA-2 K tubes for hematological assessment (0.5 mL), into tubes containing 3.2 % sodium citrate for plasma separation (1 mL), and into tubes without anticoagulant for serum preparation (1.5 mL). The parameters measured were as follows: hematological parameters, including total white blood cell (WBC) count, total red blood cell (RBC) count, hemoglobin, hematocrit, mean corpuscular volume, mean corpuscular hemoglobin, mean corpuscular hemoglobin concentration, platelet count, reticulocyte count absolute, reticulocyte count relative, neutrophil count absolute, neutrophil count relative, eosinophil count absolute, eosinophil count relative, basophil count absolute, basophil count relative, monocyte count absolute, monocyte count relative, lymphocytes absolute, lymphocytes relative, large unstained cells absolute, and large unstained cells relative; coagulation parameters, including prothrombin time and activated partial thromboplastin time; and clinical parameters, including glucose, BUN, creatinine (CREA), total protein (TP), albumin, albumin/globulin ratio, total cholesterol, triglyceride, phospholipid, AST, ALT, total bilirubin, AP, gamma-glutamyl transpeptidase (GGT), creatine phosphokinase, calcium, inorganic phosphorus, sodium, potassium and chloride.

#### 2.6.2 Urinalysis and urine chemistry

Animals in the cage were separated and transferred to a metabolic cage or a cage equipped with a device for collection of urine on the day before administration as well as terminal sacrifice (day 29), and recovery sacrifice (day 43). Before urine collection, animals were fasted overnight; however, drinking water was made available. The urine volume was recorded using a measuring cylinder, and the following parameters were measured using a Cobas U411 urine analyzer (Roche, Switzerland) using a urine reagent strip: color, clarity, pH, specific gravity, bilirubin, proteins, urobilinogen level, nitrite level, glucose level, erythrocyte count, ketone, leukocyte count, urine potassium, urine chloride, urine sodium, urine cast and epithelial cell count.

### 2.7 Necropsy and histopathologic examination

#### 2.7.1 Gross observation and organ weight

Pre-anesthesia was induced in all animals using ketamine (11−12 mg/kg) and xylazine (2−3 mg/kg) on the day of the terminal sacrifice (day 29) and recovery sacrifice (day 43) after fasting for at least 16 h. Following this, the animals were heavily sedated with thiopental sodium (75−80 mg/kg) administered intravenously and then euthanized by exsanguination. Abnormalities in the external as well as in the abdominal, thoracic, and cranial cavities were observed by a veterinary pathologist, and full macroscopic examinations were recorded.

The following organs were weighed prior to fixation: the brain, pituitary gland, liver with gall bladder, spleen, heart, thymus, salivary glands, seminal vesicles, prostate, kidneys, adrenal glands, testes, epididymis, lungs, thyroid, uterus with cervix, and ovaries. Paired organs were weighed together.

#### 2.7.2 Histopathology

Histopathological examination was conducted for the following tissues: abnormal lesions, adrenal glands, animal ID, aorta (thoracic), brain, cecum, colon, duodenum, epididymis, esophagus, eyes with optic nerve, femur with marrow, heart, ileum, jejunum, kidneys, liver with gall bladder, lung with bronchi, mammary gland, uterus with cervix, vagina, injection sites, pancreas, prostate, pituitary gland, rectum, salivary glands, sciatic nerve, seminal vesicles, skeletal muscles, skin, thoracic spinal cord, spleen, sternum with marrow, stomach, testes, thymus, thyroids, tongue, trachea, urinary bladder, mesenteric lymph node, ovaries, and mandibular lymph nodes. The tissues from each animal were preserved in 10 % neutral buffered formalin, except the eyes with the attached optic nerve, which were fixed in Davidson’s fixative, and the testes and epididymides, which were fixed in Bouin’s fixative. Especially for the lung and urinary bladder, formalin was infused and fixed. After approximately 24–72 h of fixation, tissues preserved in formalin were then placed in 70 % ethanol. Fixed tissues were embedded in paraffin, sectioned (2.5 μm), and stained with hematoxylin and eosin. Images were collected at ×200 or ×400 magnification, and microscopic evaluation was performed by a veterinary pathologist.

### 2.8 Toxicokinetic evaluation

#### 2.8.1 Bioanalysis

Approximately 1.0 mL of blood was collected from the jugular vein or vena cava on Day 1 and Week 4 from all available animals excluding the recovery animals. For vehicle control group, blood will be collected at pre-dose (0) and approximately 2 h after dosing (total 2 points). For treatment groups, blood will be collected at pre-dose (0), approximately 0.5, 1, 2, 4, 6, 10 and 24 h after dosing (total 8 points). The blood will be collected into blood collecting tubes containing potassium salt of EDTA. Blood samples will be mixed gently and placed on crushed wet-ice/kryorack and then centrifuged (approximately 3000 rpm, 10 min, 4 °C). Following centrifugation, concentration of diclofenac sodium salt (DSS) in the plasma samples was analyzed according to the validated biological sample analysis method using LC-MS/MS (KIT Study No. G218052). The obtained samples were conducted protein removal using a methanol solution containing an internal standard (Amlodipine besylate) and centrifuged. A calibration curve was created with the concentration of DSS as the x-axis and the peak area ratio of DSS and the internal standard as the y-axis using 1/x2 weighted regression. The range of the diclofenac calibration curve was 10−4000 ng/mL.

#### 2.8.2 Toxicokinetic analysis

Non-compartmental method based on blood concentration curves was used for toxicokinetic analysis. Maximum plasma concentration (C_max_) and Time to reach C_max_ (T_max_) were taken, and elimination rate constant (K_el_) elimination half-life (T_1/2_) were calculated from the plasma concentration versus time profile. In addition, area under plasma concentration-time curve (AUC) at the last quantifiable time point (AUC_last_), actual volume of distribution at steady state (V_ss_) and actual clearance (CL) were calculated using linear trapezoidal rule. All these toxicokinetic parameters were calculated and analyzed using the Phoenix® WinNonlin® (version 8.1, Centara Inc., USA)).

### 2.9 Statistical analysis

All data were statistically analyzed using the Pristima software (Version 7.4, Xybion Medical Systems Corporation, Lawrenceville, New Jersey, USA). Multiple comparison tests were performed to compare different dose groups. The data were analyzed for homogeneity of variance using Bartlett’s test. Homogeneous data were analyzed using analysis of variance, and the significance of inter-group differences was analyzed using Dunnett’s test. Heterogeneous data were analyzed using the Kruskal-Wallis test, and the significance of inter-group differences between the vehicle control and treated groups was assessed using Dunn’s rank sum test. After performing the F-test for assessing homogeneity variance between the vehicle control and recovery groups, Student’ s *t*-test was conducted to analyze significant differences between the homogeneous data of groups. The Wilcoxon rank-sum test was used for assessing differences between heterogeneous data of groups. A p value less than 0.05 was considered statistically significant

## 3 Results

### 3.1 Formulation analysis

Solutions of DSS in the range of 1−50 mg/mL were shown to be stable for 7 days under storage at room temperature in the dark. The low-, middle-, and high-dose DSS solutions, assessed at the start of dosing (week 1) and also at the last week (week 4), were found to have homogeneous distribution of DSS with coefficients of variation (CVs) within 10 % (0.3−0.9%). The concentration analysis showed that acceptable stability was within 15 % CV (101.6–102.9 % on Day 1 and 97.0–102.7 % on week 4).

### 3.2 Body weight

**Fig. 1:**
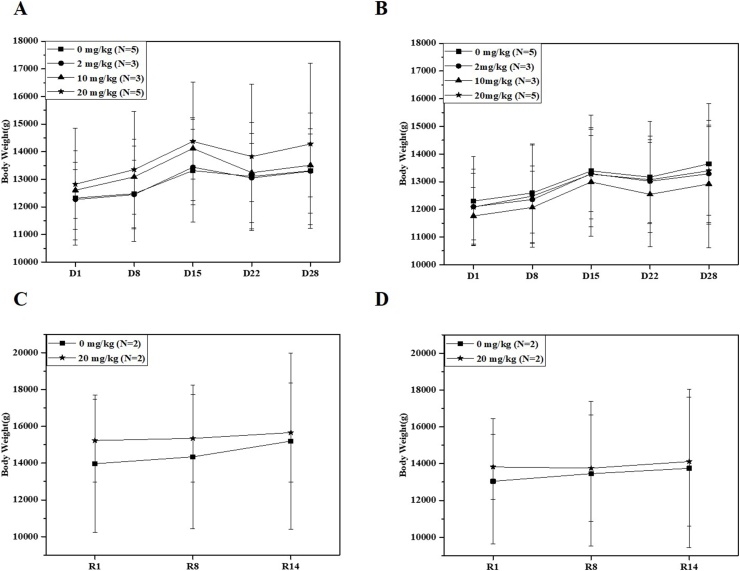
Changes in the mean body weight (± SD) of males (A) and females (B) in all four experimental groups during diclofenac sodium salt intramuscular administration period. Changes in the mean body weight of males (C) and females (D) in the vehicle (0 mg/kg) and high dose (20 mg/kg) groups during the recovery period.

As shown in Fig. 1, overall, body weight showed a tendency to increase during the administration and recovery periods. Body weight decreased slightly after 3 weeks of administration, but this decrease was not statistically significant.

### 3.3 Food consumption

In both sexes, there were no changes in food consumption following DSS administration

### 3.4 Ophthalmoscopy and electrocardiography

No DSS related changes in ophthalmoscopy and electrocardiography following DSS administration were observed.

### 3.5 Observations: survival and clinical signs

Following daily DSS administration, all minipigs were examined twice daily for clinical signs related to DSS administration using a standard operating procedure. Table 1 shows the number of minipigs showing clinical signs related to DSS administration according to group. No animals died as a result of DSS treatment during the study.

**Table 1:** The number of minipigs showing abnormal findings in clinical observation following administered DSS intramuscularly for 4 weeks and 2-week recovery period.

		Male				Female			
		Treatment			Recovery	Treatment			Recovery
		2 mg/kg	10 mg/kg	20 mg/kg	20 mg/kg	2 mg/kg	10 mg/kg	20 mg/kg	20 mg/kg
**Limping**		**2**	**1**	**1**	–	–	–	–	–
**Vomiting**		–	**3**	**5**	–	**1**	**3**	**5**	–
**Discolored urine**		–	**2**	**3**	–	–	–	**2**	–
**Swollen**	**Forelimb**	**1**	–	**1**	–	–	–	–	–
	**Hindlimb**	**2**	–	–	–	–	–	–	–
	**Pinna**	**1**	–	–	–	–	–	–	–
	**neck**	–	**2**	**3**	**1**	–	**1**	**3**	–
	**palate**	–	–	**5**	**2**	–	–	**3**	**1**
**Loss of tooth**		–	–	**3**	**2**	–	–	**1**	**1**
**Abscesses**	**Forelimb**	–	–	**1**	–	–	–		–
	**Hindlimb**	**1**	**1**	–	–	–	–	**1**	–
**Ulceration**	**Forelimb**	–	–	**1**	–	–	–		–
	**Pinna**	–	**2**	–	–	–	–	**1**	–
	**Oral region**	–	–	**1**	–	–	–		–

Low-dose group (2 mg/kg/day): Slight emesis was observed after 13–14 days of administration in one female minipig. Two male minipigs had consistent swelling (slight to moderate) of the lower forelimb, hindlimb, or pinna from day 5 of administration; in addition, these animals showed limping from day 16 to the day of necropsy. In one male animal, slight abscess was noted in the lower hindlimb from day 22 to the day of necropsy.

Middle-dose group (10 mg/kg/day):

Emesis (slight to moderate) was sporadically observed after day 11. Limping was showed from at day 16 that persisted until the day of necropsy. In one male minipig, abscess of the lower hindlimb (slight to moderate) was observed from day 16 to the day of necropsy. A slight swelling of the neck was noted in two males and one female minipigs from day 18 to the day of necropsy. In two male animals, some amount of blood was noted intermittently in the urine after 2 weeks after administration. Ulcerations in the pinnae (slight to severe) appeared in two males at day 10 and were continuously present until the day of necropsy.

High-dose group (20 mg/kg/day):

Chronic intermittent emesis (slight to moderate) was observed in all animals from day 7. One male developed a limp at day 16 that persisted until the day of necropsy. There was a slight swelling in the necks and lower forelimbs in both sexes (3/5, 3/5) from day 17 to the day of necropsy after the recovery period. Slight abscesses in the lower forelimbs and hindlimbs were observed in one male and one female for 2–3 days after 20 days of administration. Ulcerations (slight to moderate) of the lower forelimbs, upper/lower oral regions, and the pinnae were noted in two male minipigs and one female minipig from day 11 to the day of necropsy. In addition, swelling of the palate (slight to moderate) was observed in all male and in three female minipigs from day 18 to the day of necropsy day after the recovery period Blood was sporadically noted in the urine of three male and two female minipigs (slight to moderate) after 2 weeks of administration. Tooth loss occurred in three male animals and one female animal from day 18.

### 3.6 Clinical pathology

Table 2 shows the hematology and coagulation parameters that were statistically significantly different in minipigs pre- and post-administration of DSS, and during the subsequent 2-week recovery period. Compared with those in the vehicle control group, males from the middle-dose group and females in the high-dose group receiving 10 and 20 mg/kg/day at day 29 had significant (p < 0.05−0.01) decreases in mean RBC counts (0.53-fold), hemoglobin level (0.56-fold), and hematocrit level (0.61-fold), as well as significant (p < 0.05−0.01) increases in WBC (2.55-fold), absolute and relative neutrophil (4.12-fold and 1.68-fold, respectively), and absolute monocyte (2.62-fold) counts. Moreover, significant (p < 0.05−0.01) increases in mean corpuscular volume (1.15-fold), absolute and relative reticulocyte counts (5.57-fold and 8.66-fold), and platelet counts (2.21-fold) were noted in both sexes in the high-dose group receiving 20 mg/kg/day on day 29. In addition, the absolute eosinophil count significantly (p < 0.05) increased (3.76-fold) in males in the high-dose group on day 29. These changes were partially or fully resolved after the recovery period.

**Table 2:** Summary of selected hematological parameters in minipigs administered DSS intramuscularly for 4 weeks.

Sex		Male					Female			
Dose (mg/kg/day)		0	2	10	20	0		2	10	20
**RBC** **(x10^6/uL)**	**Pre-treatment**	7.44 ± 0.696	7.16 ± 0.591	7.02 ± 0.280	7.26 ± 0.627	6.99 ± 0.661		6.82 ± 0.528	6.87 ± 0.205	7.16 ± 0.325
	**Treatment**	8.29 ± 0.960	7.48 ± 0.401	6.36*D ± 0.569	4.39**D ± 0.799	7.40 ± 0.552		7.60 ± 0.582	7.20 ± 0.447	5.05**D ± 0.745
	**Recovery**	8.25 ± 0.785			6.91 ± 0.106	8.14 ± 0.460				6.57*T ± 0.212
**HGB** **(g/dL)**	**Pre-treatment**	12.5 ± 0.59	12.2 ± 1.15	12.1 ± 0.45	12.2 ± 0.69	12.1 ± 0.57		11.8 ± 0.72	11.5 ± 0.55	12.2 ± 0.37
	**Treatment**	14.2 ± 0.81	12.8 ± 1.03	11.3**D ± 0.89	8.0**D ± 1.42	13.0 ± 0.87		13.4 ± 1.40	12.8 ± 1.39	9.7**D ± 1.08
	**Recovery**	14.6 ± 0.14			12.3**T ± 0.07	14.4 ± 0.71				12.9 ± 0.14
**HCT** **(%)**	**Pre-treatment**	40.4 ± 2.13	39.4 ± 4.57	38.7 ± 0.96	38.5 ± 3.29	38.2 ± 2.72		37.6 ± 2.85	36.2 ± 1.77	39.3 ± 0.94
	**Treatment**	44.6 ± 2.88	40.8 ± 2.91	36.3*D ± 2.27	27.0**D ± 4.2	41.9 ± 2.87		43.5 ± 4.00	40.9 ± 4.31	32.6**D ± 3.59
	**Recovery**	44.2 ± 1.06			40.5*T ± 0.14	46.5 ± 3.75				42.3 ± 0.00
**MCV** **(fL)**	**Pre-treatment**	54.5 ± 3.02	54.9 ± 2.12	55.2 ± 0.87	53.2 ± 1.63	54.8 ± 2.56		55.2 ± 0.53	52.7 ± 3.66	55.0 ± 2.43
	**Treatment**	54.0 ± 2.87	54.5 ± 1.50	57.2 ± 1.61	61.8**D ± 3.83	56.7 ± 1.61		57.2 ± 1.19	56.8 ± 5.58	65.0*D ± 5.68
	**Recovery**	53.9 ± 6.43			58.6 ± 1.13	57.1 ± 1.34				64.5 ± 2.05
**MCH** **(pg)**	**Pre-treatment**	16.8 ± 1.12	17.0 ± 0.50	17.3 ± 0.12	16.8 ± 0.81	17.4 ± 1.27		17.4 ± 0.53	16.7 ± 1.01	17.1 ± 0.81
	**Treatment**	17.2 ± 1.10	17.0 ± 0.71	17.8 ± 0.26	18.3 ± 1.29	17.6 ± 0.66		17.7 ± 0.61	17.9 ± 1.72	19.4 ± 1.47
	**Recovery**	17.8 ± 1.84			17.7 ± 0.21	17.7 ± 0.07				19.7*T ± 0.42
**MCHC** **(g/dL)**	**Pre-treatment**	30.9 ± 0.50	31.0 ± 0.76	31.2 ± 0.35	31.6 ± 0.97	31.8 ± 1.16		31.5 ± 1.11	31.7 ± 0.32	31.0 ± 0.29
	**Treatment**	31.9 ± 0.94	31.2 ± 0.45	31.1 ± 0.38	29.6**D ± 1.37	31.1 ± 0.46		30.9 ± 0.42	31.5 ± 0.25	29.8**D ± 0.55
	**Recovery**	33.1 ± 0.57			30.2*T ± 0.35	31.0 ± 0.99				30.6 ± 0.28
**RET%** **(%)**	**Pre-treatment**	1.06 ± 0.483	1.03 ± 0.040	1.44 ± 0.397	1.28 ± 0.586	1.15 ± 0.418		0.99 ± 0.068	1.30 ± 0.457	0.97 ± 0.323
	**Treatment**	1.04 ± 0.422	0.55 ± 0.114	2.08 ± 1.156	6.87*R ± 3.670	0.98 ± 0.438		1.32 ± 0.725	1.79 ± 0.531	8.49**R ± 5.224
	**Recovery**	0.89 ± 0.318			2.59 ± 1.025	1.95 ± 0.035				0.83**T ± 0.148
**RETA** **(10^9/L)**	**Pre-treatment**	77 ± 27.4	74 ± 8.8	102 ± 31.4	93 ± 45.4	79 ± 29.4		68 ± 6.6	90 ± 30.5	69 ± 22.4
	**Treatment**	85 ± 31.2	41 ± 8.7	128 ± 61.0	290 ± 127.7	74 ± 36.7		102 ± 59.2	129 ± 37.2	412**R ± 221.1
	**Recovery**	72 ± 19.4			178 ± 68.0	158 ± 12.3				54**T ± 11.3
**PLT** **(10^3/uL)**	**Pre-treatment**	540.0 ± 35.26	565.0 ± 16.52	644.7 ± 87.31	558.2 ± 143.09	577.0 ± 114.36		565.7 ± 50.29	596.0 ± 195.60	628.8 ± 85.20
	**Treatment**	465.6 ± 19.39	576.0 ± 63.55	775.0 ± 155.78	973.6**R ± 122.86	507.4 ± 222.78		543.0 ± 1.00	703.3 ± 83.03	1123.6*R ± 107.26
	**Recovery**	392.5 ± 23.33			1002.0**T ± 2.83	319.0 ± 326.68				863.5 ± 102.53
**NEU%** **(%)**	**Pre-treatment**	44.5 ± 9.83	51.7 ± 15.03	47.4 ± 8.81	43.7 ± 7.57	39.6 ± 14.17		36.8 ± 10.68	42.5 ± 2.25	31.9 ± 6.32
	**Treatment**	42.2 ± 6.16	41.7 ± 3.90	69.4**D ± 3.17	67.8**D ± 10.51	36.0 ± 3.28		39.6 ± 7.33	53.1*D ± 6.61	60.3**D ± 8.32
	**Recovery**	41.8 ± 0.07			42.9 ± 8.49	22.9 ± 4.31				48.4 ± 20.58
**LYM%** **(%)**	**Pre-treatment**	49.4 ± 8.98	44.0 ± 14.32	46.6 ± 7.96	51.4 ± 7.25	54.0 ± 15.39		57.2 ± 9.96	51.8 ± 2.57	62.1 ± 5.65
	**Treatment**	50.2 ± 5.31	51.5 ± 4.76	23.4**D ± 3.60	24.6**D ± 6.72	56.0 ± 3.13		54.6 ± 6.87	35.1**D ± 8.57	31.9**D ± 6.70
	**Recovery**	51.0 ± 0.42			50.2 ± 9.62	70.2 ± 4.95				45.1 ± 16.90
**BAS%** **(%)**	**Pre-treatment**	0.3 ± 0.12	0.2 ± 0.12	0.3 ± 0.12	0.3 ± 0.08	0.2 ± 0.05		0.7 ± 0.70	0.3 ± 0.10	0.4 ± 0.05
	**Treatment**	0.4 ± 0.19	0.5 ± 0.44	0.2 ± 0.00	0.2*R ± 0.07	0.4 ± 0.09		0.3 ± 0.00	0.3 ± 0.10	0.2**R ± 0.05
	**Recovery**	0.6 ± 0.14			0.5 ± 0.21	0.5 ± 0.14				0.4 ± 0.14
**WBC** **(x10^3/uL)**	**Pre-treatment**	12.50 ± 2.253	18.43 ± 3.837	15.11 ± 4.818	13.15 ± 3.150	14.68 ± 3.040		12.68 ± 2.159	13.03 ± 1.343	11.89 ± 2.534
	**Treatment**	14.70 ± 3.588	17.95 ± 2.207	36.20**D ± 11.285	37.46**D ± 9.516	14.13 ± 1.663		14.39 ± 1.178	25.15 ± 10.446	31.03*R ± 2.980
	**Recovery**	10.32 ± 1.527			16.64 ± 2.885	11.60 ± 0.255				17.80 ± 3.048
**NEUA** **(x10^3/uL)**	**Pre-treatment**	5.70 ± 2.221	9.91 ± 4.434	7.36 ± 3.121	5.89 ± 2.372	5.81 ± 2.530		4.60 ± 1.278	5.52 ± 0.304	3.74 ± 0.882
	**Treatment**	6.32 ± 2.438	7.52 ± 1.534	25.37*R ± 9.094	26.06**R ± 9.209	5.05 ± 0.402		5.73 ± 1.409	13.35 ± 5.624	18.88**R ± 4.141
	**Recovery**	4.31 ± 0.622			7.26 ± 2.652	2.66 ± 0.559				8.92 ± 5.134
**LYMA** **(x10^3/uL)**	**Pre-treatment**	6.06 ± 0.960	7.74 ± 0.690	6.85 ± 1.778	6.64 ± 1.090	7.91 ± 3.047		7.34 ± 2.372	6.76 ± 0.958	7.42 ± 2.014
	**Treatment**	7.27 ± 1.210	9.19 ± 0.714	8.21 ± 1.296	8.79 ± 1.419	7.93 ± 1.212		7.83 ± 0.898	8.31 ± 1.605	9.74 ± 1.097
	**Recovery**	5.27 ± 0.820			8.22*T ± 0.148	8.14 ± 0.389				7.76 ± 1.633
**MONA** **(x10^3/uL)**	**Pre-treatment**	0.55 ± 0.153	0.50 ± 0.045	0.63 ± 0.172	0.40 ± 0.072	0.73 ± 0.446		0.47 ± 0.142	0.46 ± 0.217	0.53 ± 0.228
	**Treatment**	0.73 ± 0.197	0.74 ± 0.214	1.91*R ± 0.984	1.39*R ± 0.219	0.66 ± 0.215		0.56 ± 0.046	0.74 ± 0.318	1.28**D ± 0.339
	**Recovery**	0.54 ± 0.007			0.56 ± 0.134	0.44 ± 0.141				0.57 ± 0.035
**EOSA** **(x10^3/uL)**	**Pre-treatment**	0.03 ± 0.024	0.07 ± 0.085	0.03 ± 0.023	0.04 ± 0.031	0.05 ± 0.035		0.02 ± 0.010	0.04 ± 0.021	0.03 ± 0.011
	**Treatment**	0.21 ± 0.077	0.28 ± 0.049	0.35 ± 0.061	0.79*R ± 0.751	0.32 ± 0.244		0.16 ± 0.017	2.47 ± 3.546	0.71 ± 0.437
	**Recovery**	0.08 ± 0.042			0.49 ± 0.262	0.23 ± 0.092				0.38 ± 0.417

The values are expressed as mean ± SD, *D: Dunnett LSD Test significant at the 0.05, **D: Dunnett LSD Test significant at the 0.01, *R: Dunn Rank Sum Test Significant at the 0.05, **R: Dunn Rank Sum Test Significant at the 0.01, *T: *t*-test Significant at the 0.05, **T: *t*-test Significant at the 0.01.

RBC, total red blood cell count; HGB, hemoglobin; HCT, hematocrit; MCV, mean corpuscular volume; MCH, mean corpuscular hemoglobin; MCHC, mean corpuscular hemoglobin concentration; RET%, reticulocyte count relative; RETA, reticulocyte count absolute; PLT, platelet count; NEU%, neutrophils relative; LYM%, lymphocytes relative; BAS%, basophils relative; WBC, total leukocyte count; NEUA, neutrophils absolute; LYMA, lymphocytes absolute; MONA, monocytes absolute; EOSA, eosinophils absolute.

The clinical chemistry parameters that were statistically significantly changed are shown in Table 3, along with the mean values and standard deviations. There were significant (p < 0.05−0.01) decreases in the TP (0.62-fold), albumin (0.56-fold), GGT (0.63-fold), AP (0.54-fold), and calcium (0.8-fold) levels in both sexes in the high-dose group on day 29. Compared with those in the vehicle control group, males in the middle-dose group and females in the high-dose group had decreased ALT levels and males in the middle-dose group had decreased CREA levels on day 29. These were partially or fully resolved after the recovery period.

**Table 3:** Summary of selected clinical chemistry parameters in minipigs administered DSS intramuscularly for 4 weeks.

Sex		Male				Female			
Dose (mg/kg/day)		0	2	10	20	0	2	10	20
**CREA** **(mg/dL)**	**Pre-treatment**	1.07 ± 0.087	1.15 ± 0.072	1.09 ± 0.046	1.07 ± 0.133	1.37 ± 0.703	1.02 ± 0.211	0.97 ± 0.103	1.02 ± 0.056
	**Treatment**	1.02 ± 0.104	0.97 ± 0.035	0.73**D ± 0.101	0.70**D ± 0.079	0.98 ± 0.093	1.01 ± 0.081	0.95 ± 0.159	0.78 ± 0.154
	**Recovery**	0.90 ± 0.049			1.00 ± 0.071	1.12 ± 0.078			1.01 ± 0.177
**TP** **(g/dL)**	**Pre-treatment**	7.18 ± 0.316	6.67 ± 0.270	7.31 ± 0.235	6.98 ± 0.215	6.62 ± 0.482	6.86 ± 0.248	6.81 ± 0.102	6.69 ± 0.265
	**Treatment**	7.92 ± 0.275	7.61 ± 0.231	7.68 ± 0.561	5.19**R ± 1.215	7.61 ± 0.298	7.68 ± 0.158	6.69 ± 0.462	4.72**D ± 0.844
	**Recovery**	7.80 ± 0.361			5.72*T ± 0.516	7.09 ± 0.820			7.21 ± 0.431
**ALB** **(g/dL)**	**Pre-treatment**	4.22 ± 0.198	4.11 ± 0.131	4.28 ± 0.154	4.12 ± 0.108	4.00 ± 0.226	3.99 ± 0.040	3.99 ± 0.091	4.05 ± 0.148
	**Treatment**	4.37 ± 0.149	3.95 ± 0.240	3.60 ± 0.675	2.45**D ± 0.546	4.15 ± 0.125	4.25 ± 0.163	3.66*D ± 0.140	2.39**D ± 0.370
	**Recovery**	4.29 ± 0.014			3.12**T ± 0.007	4.06 ± 0.290			3.45 ± 0.035
**A/G** **(ratio)**	**Pre-treatment**	1.43 ± 0.081	1.62 ± 0.195	1.41 ± 0.017	1.45 ± 0.107	1.54 ± 0.128	1.40 ± 0.120	1.42 ± 0.104	1.54 ± 0.095
	**Treatment**	1.24 ± 0.068	1.08 ± 0.076	0.93 ± 0.38	0.95 ± 0.280	1.21 ± 0.067	1.23 ± 0.050	1.22 ± 0.086	1.04*D ± 0.117
	**Recovery**	1.23 ± 0.113			1.23 ± 0.247	1.35 ± 0.141			0.93 ± 0.120
**ALT** **(IU/L)**	**Pre-treatment**	54.8 ± 6.39	48.4 ± 3.95	42.5 ± 2.63	54.5 ± 11.06	53.9 ± 16.14	45.8 ± 4.15	56.9 ± 9.80	46.8 ± 3.16
	**Treatment**	73.8 ± 11.71	70.8 ± 19.87	43.7**D ± 2.23	28.7**D ± 6.38	55.0 ± 5.24	70.0 ± 3.33	70.2 ± 13.51	26.1**D ± 10.34
	**Recovery**	70.3 ± 17.25			35.6 ± 4.17	50.9 ± 7.99			41.9 ± 2.76
**GGT** **(IU/L)**	**Pre-treatment**	65.89 ± 4.813	65.70 ± 7.034	69.65 ± 8.979	65.19 ± 4.526	61.51 ± 6.130	62.20 ± 11.054	69.97 ± 9.700	64.70 ± 6.497
	**Treatment**	61.00 ± 2.497	58.70 ± 5.256	61.68 ± 10.429	38.59**D ± 6.670	64.14 ± 5.459	63.21 ± 6.433	62.36 ± 2.934	42.03**D ± 10.052
	**Recovery**	50.33 ± 3.677			58.52 ± 2.510	65.66 ± 14.128			63.47 ± 9.214
**ALP** **(IU/L)**	**Pre-treatment**	367.8 ± 37.19	365.2 ± 45.88	356.1 ± 21.50	353.5 ± 50.22	400.2 ± 103.37	330.4 ± 50.84	320.6 ± 24.28	338.5 ± 17.46
	**Treatment**	280.4 ± 49.27	257.4 ± 53.64	208.7 ± 59.76	150.1**D ± 25.70	289.3 ± 21.44	300.8 ± 54.51	271.5 ± 112.20	162.1*R ± 27.49
	**Recovery**	239.6 ± 59.40			315.1 ± 27.65	286.8 ± 87.54			253.8 ± 61.59
**TCHO** **(mg/dL)**	**Pre-treatment**	101.6 ± 6.69	87.0*D ± 3.4	93.0 ± 11.36	85.2**D ± 3.56	84.8 ± 16.51	97.0 ± 21.66	84.7 ± 24.79	97.4 ± 16.56
	**Treatment**	100.4 ± 8.38	86.7 ± 4.51	71.7**D ± 9.29	81.2**D ± 9.15	101.4 ± 9.79	102.7 ± 15.31	81.7 ± 12.06	91.4 ± 9.58
	**Recovery**	103.0 ± 8.49			93.0 ± 4.24	90.0 ± 15.56			104.0 ± 5.66
**TG** **(mg/dL)**	**Pre-treatment**	41.7 ± 13.97	22.9 ± 11.27	27.2 ± 2.39	27.8 ± 9.38	24.8 ± 12.45	32.8 ± 9.51	19.2 ± 3.55	26.2 ± 3.69
	**Treatment**	36.2 ± 12.86	31.1 ± 1.36	28.6 ± 3.61	68.8 ± 28.37	34.3 ± 8.38	41.3 ± 11.55	39.8 ± 20.42	82.0**D ± 25.64
	**Recovery**	30.9 ± 22.34			38.9 ± 10.61	83.4 ± 40.87			34.5 ± 0.07
**Ca** **(mg/dL)**	**Pre-treatment**	10.86 ± 0.301	10.50 ± 0.641	10.92 ± 0.150	10.46 ± 0.511	10.64 ± 0.472	10.96 ± 0.281	10.45 ± 0.030	11.06 ± 0.297
	**Treatment**	11.20 ± 0.217	10.63 ± 0.152	10.36 ± 0.592	9.00**D ± 0.590	10.97 ± 0.272	11.10 ± 0.222	10.48 ± 0.071	9.24**D ± 0.289
	**Recovery**	10.99 ± 0.332			9.87*T ± 0.085	10.71 ± 0.212			10.31 ± 0.141
**IP** **(mg/dL)**	**Pre-treatment**	8.74 ± 0.370	8.08 ± 0.409	8.71 ± 0.116	7.88 ± 1.501	8.17 ± 0.533	7.72 ± 0.350	7.76 ± 0.781	8.22 ± 0.465
	**Treatment**	7.82 ± 0.326	7.29 ± 0.263	8.07 ± 0.070	7.74 ± 0.420	7.76 ± 0.143	7.64 ± 0.431	8.34 ± 0.611	8.97*D ± 0.803
	**Recovery**	7.37 ± 0.057			7.99 ± 0.658	7.02 ± 0.417			7.89 ± 0.629
**K** **(mmol/L)**	**Pre-treatment**	3.97 ± 0.306	3.73 ± 0.076	4.24 ± 0.539	3.73 ± 0.296	4.02 ± 0.587	4.13 ± 0.596	3.77 ± 0.125	4.05 ± 0.550
	**Treatment**	5.23 ± 0.432	4.62 ± 0.473	4.73 ± 0.436	4.57 ± 0.472	4.88 ± 0.490	4.73 ± 0.257	5.56 ± 0.116	4.96 ± 0.679
	**Recovery**	4.93 ± 0.544			4.48 ± 0.325	4.81 ± 0.057			4.43*T ± 0.035
**PL** **(mg/dL)**	**Pre-treatment**	121.0 ± 16.02	103.7 ± 1.53	101.7 ± 8.74	104.0 ± 6.82	96.2 ± 28.01	127.0 ± 30.12	99.3 ± 20.50	110.6 ± 21.17
	**Treatment**	122.6 ± 10.64	107.3 ± 8.14	74.0**D ± 12.12	94.6**D ± 13.24	122.0 ± 10.02	129.7 ± 13.32	108.3 ± 21.20	110.6 ± 7.50
	**Recovery**	128.5 ± 6.36			112.0 ± 16.97	104.5 ± 13.44			109.5 ± 2.12

The values are expressed as mean ± SD, *D: Dunnett LSD Test significant at the 0.05. **D: Dunnett LSD Test significant at the 0.01, *R: Dunn Rank Sum Test Significant at the 0.05, **R: Dunn Rank Sum Test Significant at the 0.01, *T: *t*-test Significant at the 0.05, **T: *t*-test Significant at the 0.01.

CREA, creatinine; TP, total protein; ALB, albumin; A/G, albuminglobulin ratio; ALT, alanine aminotransferase; GGT, gamma glutamyl transpeptidase; ALP, alkaline phosphatase; TCHO, total cholesterol; TG, triglyceride; Ca, calcium; IP, inorganic phosphorus; K, potassium; PL, phospholipid.

Selected urinalysis and urine sediment data are shown in Table 4. In the urinalysis, a score of 5 or greater was observed for males in the middle-dose group (2/3 and 2/5, respectively) and for females in the high-dose group (4/5). The urine sediment examination revealed the presence of RBCs and WBCs in the urine of males in the middle-dose group over (1/3 and 1/5, respectively) and of females in the high-dose group (3/5). Other statistically significant changes were not considered DSS-related symptoms as they did not show a dose-dependent effect.

**Table 4:** Summary of selected urinalysis and urine sediment parameters in minipigs administered DSS intramuscularly for 4 weeks.

sex		Male						Female					
Dose (mg/kg/day)	Animal No.	Treatment			Recovery			Treatment			Recovery		
		ERY	URBC	UWBC	ERY	URBC	UWBC	ERY	URBC	UWBC	ERY	URBC	UWBC
**0**	**1**	**Neg**	**<1**	**<1**				**Neg**	**<1**	**<1**			
	**2**	**Neg**	**<1**	**<1**				**Neg**	**<1**	**<1**			
	**3**	**Neg**	**<1**	**<1**				**Neg**	**<1**	**<1**			
	**4**	**Neg**	**<1**	**<1**	**Neg**	**<1**	**<1**	**Neg**	**<1**	**<1**	**Neg**	**<1**	**<1**
	**5**	**Neg**	**<1**	**<1**	**Neg**	**<1**	**<1**	**Neg**	**1−4**	**<1**	**Neg**	**<1**	**<1**
**2**	**6**	**3+**	**1−4**	**1−4**				**Neg**	**<1**	**<1**			
	**7**	**4+**	**5−9**	**1−4**				**Neg**	**1−4**	**1−4**			
	**8**	**Neg**	**<1**	**<1**				**2+**	**1−4**	**<1**			
**10**	**9**	**4+**	**10−29**	**1−4**				**3+**	**5−9**	**<1**			
	**10**	**5+**	**1−4**	**1−4**				**Neg**	**<1**	**<1**			
	**11**	**5+**	**>1/2**	**1−4**				**Neg**	**<1**	**<1**			
**20**	**12**	**2+**	**1−4**	**10−29**				**2+**	**1−4**	**1−4**			
	**13**	**Neg**	**1−4**	**1−4**				**5+**	**10−29**	**5−9**			
	**14**	**1+**	**1−4**	**5−9**				**5+**	**>30**	**1−4**			
	**15**	**5+**	**10−29**	**1−4**	**Neg**	**1−4**	**1−4**	**5+**	**>1/2**	**<1**	**Neg**	**<1**	**1−4**
	**16**	**5+**	**5−9**	**>30**	**Neg**	**1−4**	**1−4**	**5+**	**5−9**	**1−4**	**Neg**	**<1**	**1−4**

ERY, erythrocyte; URBC, red blood cell in urine; white blood cell in urine; Neg, negative.

### 3.7 Histopathology

#### 3.7.1 Macroscopic examination

Table 5 summarizes the number of animals showing abnormal findings on macroscopic examination. Discoloration (brown, red, or yellow) at the injection site was observed in all animals except the vehicle control group at the end of the administration. A female in the vehicle control group and all animals in the high-dose group showed discoloration at the injection site after the recovery period. A swollen ear (1/3 males) or swollen lower hindlimbs (1/3 male and 1/3 female) was observed in the low-dose group receiving 2 mg/kg/day. Ulceration was noted around the mouth in the high-dose group receiving 20 mg/kg/day (3/3 males), and ulceration of the ear was noted in the middle-dose (2/3 males) and high-dose (1/3 female) groups receiving 10 and 20 mg/kg/day, respectively. In addition, ulceration of the lower forelimb was observed in the middle-dose (1/3 male) and high-dose (1/3 male) groups receiving 10 and 20 mg/kg/day, respectively, as well as in the lower hindlimb of the high-dose (1/3 female) group receiving 20 mg/kg/day. These findings were not observed in the recovery group and therefore appeared to be fully reversible. Enlarged and red discolored mesenteric lymph nodes and omental adhesions to the stomach were also observed in one male in the high-dose group. A cyst in the right kidney was observed in a male in the middle-dose group receiving 10 mg/kg/day. Pale discoloration of the kidneys (1/2 female) and reductions in thymus sizes (2/2 males) were observed in the high-dose recovery group receiving 20 mg/kg/day.

**Table 5:** The number of minipigs showing abnormal findings in macroscopic examination following administered DSS intramuscularly for 4 weeks.

Sex		Male						Female					
		Treatment				Recovery		Treatment				Recovery	
Dose (mg/kg/day)		0	2	10	20	0	2	0	2	10	20	0	2
Kidney	Cyst, Partial, right, clear	–	–	1	–	–	–	–	–	–	–	–	–
	Hydronephrosis, unilateral	–	–	–	1	–	–	1	–	–	–	–	–
	Discoloration, both, pale, partial	–	–	–	–	–	–	–	–	–	–	–	1
Lymph node, mesenteric	Discoloration, partial, red	–	–	–	1	–	–	–	–	–	–	–	–
	Increased size, slight	–	–	–	1	–	–	–	–	–	–	–	–
Skin	Swollen, ear, bilateral	–	1	–	–	–	–	–	–	–	–	–	–
	Swollen, lower hindlimb, unilateral	–	1	–	–	–	–	–	1	–	–	–	–
	Ulceration, around mouth, partial	–	–	–	3	–	–	–	–	–	–	–	–
	Ulceration, lower forelimb, partial, unilateral	–	–	1	–	–	–	–	–	–	–	–	–
	Ulceration, lower forelimb, partial, bilateral	–	–	–	1	–	–	–	–	–	–	–	–
	Ulceration, lower hindlimb, partial, unilateral	–	–	–	–	–	–	–	–	–	1	–	–
	Ulceration, ear, unilateral	–	–	1	–	–	–	–	–	–	–	–	–
	Ulceration, ear, partial, unilateral	–	–	1	–	–	–	–	–	–	1	–	–
Injection site(s)	Discoloration, bilateral, partial, brown	–	–	3	3	–	1	–	2	2	3	–	2
	Discoloration, bilateral, partial, red	–	2	–	–	–	–	–	1	–	–	–	–
	Discoloration, bilateral, partial, yellow	–	–	–	–	–	1	–	–	–	–	–	–
	Discoloration, unilateral, partial, brown	–	–	–	–	–	–	–	–	1	–	1	–
	Discoloration, unilateral, partial, red	–	1	–	–	–	–	1	–	–	–	–	–
Stomach	Adhesion, serosa	–	–	–	1	–	–	–	–	–	–	–	1
Lung with bronchi	Discoloration, bilateral, diaphragmatic lobe, dark, partial	–	–	–	1	–	–	–	–	–	–	–	–
	Discoloration, partial, all lobe, dark	–	1	–	–	–	–	–	–	–	–	–	–
	Discoloration, partial, left diaphragmatic lobe, dark	–	–	1	–	–	–	–	–	–	–	–	–
	Discoloration, partial, right cardial lobe, dark	–	–	–	1	–	–	–	–	–	–	–	–
	Discoloration, right diaphragmatic lobe, partial, dark-red	–	–	–	–	–	1	–	–	–	–	–	–
Thymus	Decreased size, slight, thoracic	–	–	–	–	–	2	–	–	–	–	–	–
Jejunum	Intussusception, several	–	–	–	–	–	1	–	–	–	–	–	1
	Intussusception, focal	–	–	–	–	–	–	–	–	–	–	–	1

#### 3.7.2 Organ weight

Organ weight (mean and standard deviation) are shown in Table 6. The absolute and relative (to terminal body weight) weight were significantly increased in the liver (1.59-fold and 1.72-fold, respectively) and kidney (1.93-fold and 1.79-fold) of males and females receiving 20 mg/kg/day compared with those in the vehicle control group. These changes showed could be fully resolved in the recovery phase.

**Table 6:** Summary of selected organ weight in minipigs administered DSS intramuscularly for 4 weeks.

			Treatment				Recovery	
		Dose (mg/kg/day)	0	2	10	20	0	20
		No. of animals	3	3	3	3	2	2
**Male**	**Absolute Organ Weight**	**TBW**	12408.0 ± 657.45	13008.3 ± 1605.09	13329.0 ± 1068.48	13268.7 ± 3619.87	15213.5 ± 4608.21	15487.0 ± 2351.84
		**Adrenal glands**	1.713 ± 0.795	1720 ± 0.0529	2.123 ± 0.3535	2.437**D ± 0.0666	2.005 ± 0.2333	1.985 ± 0.2475
		**Kidneys**	40.213 ± 2.2080	46.213 ± 4.4893	77.453**D ± 12.9408	74.200**D ± 3.8590	51.815 ± 15.6058	63.060 ± 2.9274
		**Liver with gall bladder**	236.523 ± 19.2786	227.817 ± 19.5324	325.417**D ± 27.6150	357.680**D ± 36.7460	292.210 ± 47.8428	348.785 ± 26.4670
		**Lung**	93.513 ± 11.3073	100.407 ± 10.8740	112.660 ± 10.6375	105.970 ± 18.5657	113.065 ± 26.8913	121.300 ± 20.4212
	**Relative Organ Weight**	**Adrenal glands**	0.0138 ± 0.00145	0.0133 ± 0.00116	0.0159 ± 0.00155	0.0193 ± 0.00505	0.0136 ± 0.00258	0.0128 ± 0.00035
		**Kidneys**	0.3244 ± 0.01709	0.3567 ± 0.03121	0.5812**D ± 0.08577	0.5809**D ± 0.12018	0.3407 ± 0.00061	0.4105 ± 0.04343
		**Liver with gall bladder**	1.9060 ± 0.11567	1.7565 ± 0.06483	2.4521 ± 0.29713	2.7776*D ± 0.46034	1.9632 ± 0.28017	2.2653 ± 0.17310
		**Lung**	0.7519 ± 0.05123	0.7795 ± 0.12880	0.8500 ± 0.12186	0.8122 ± 0.07480	0.7509 ± 0.05068	0.7822 ± 0.01307
**Female**	**Absolute Organ Weight**	**TBW**	13937.3 ± 2062.45	13184.7 ± 1390.90	13042.7 ± 2127.01	13069.7 ± 1683.05	13590.0 ± 4309.11	13923.5 ± 3049.75
		**Adrenal glands**	1.770 ± 0.2052	1.673 ± 0.1716	2.017 ± 0.1457	2.200 ± 0.2516	1.530 ± 0.1838	2.185 ± 0.0071
		**Kidneys**	54.337 ± 18.8410	42.650 ± 5.8445	54.540 ± 12.0368	70.820 ± 10.8054	42.525 ± 11.0804	65.445 ± 2.3688
		**Liver with gall bladder**	234.347 ± 36.5850	246.743 ± 29.6513	286.977 ± 63.7375	371.760*D ± 66.1142	235.645 ± 49.8722	312.305 ± 89.3005
		**Lung**	98.660 ± 16.2672	101.347 ± 13.3355	108.690 ± 20.7822	114.653 ± 5.0247	101.780 ± 16.1503	120.065 ± 25.8730
	**Relative Organ Weight**	**Adrenal glands**	0.0127 ± 0.00075	0.0129 ± 0.00241	0.0156 ± 0.00159	0.0170 ± 0.00294	0.0116 ± 0.00233	0.0161 ± 0.00357
		**Kidneys**	0.3828 ± 0.07268	0.3233 ± 0.00818	0.4157 ± 0.02501	0.5443*D ± 0.07660	0.3159 ± 0.01862	0.4797 ± 0.08805
		**Liver with gall bladder**	1.6802 ± 0.01843	1.8732 ± 0.04038	2.1933 ± 0.21403	2.8912**R ± 0.76238	1.7645 ± 0.19250	2.2262 ± 0.15375
		**Lung**	0.7072 ± 0.03088	0.7687 ± 0.02051	0.8307*D ± 0.02514	0.8840**D ± 0.08181	0.7687 ± 0.12491	0.8627 ± 0.00313

The values are expressed as mean ± SD, *D: Dunnett LSD Test significant at the 0.05, **D: Dunnett LSD Test significant at the 0.01, **R: Dunn Rank Sum Test Significant at the 0.01. TBW, terminal body weight.

#### 3.7.3 Microscopic examination

Table 7 provides the number of minipigs showing abnormal microscopic findings following administered DSS intramuscularly for 4 weeks and recovery period for 2 weeks.

**Table 7:** The number of minipigs showing abnormal findings in microscopic examination following administered DSS intramuscularly for 4 weeks and 2-week recovery period.

		Male						Female					
		Treatment			Recovery			Treatment			Recovery		
	Dose (mg/kg/day)	0	2	10	20	0	20	0	2	10	20	0	20
Adrenal glands	Infiltration, mononuclear cell	–	–	–	1	–	–	–	–	–	1	–	–
	Vacuolation, medulla	–	–	–	2	–	–	–	–	–	2	–	–
Cecum	Erosion/ulcer	–	–	–	–	–	–	–	–	–	1	–	–
	Infiltration, eosinophil, submucosa	–	–	–	1	–	–	–	–	–	1	–	–
Colon	Erosion/ulcer	–	–	–	1	–	–	–	–	–	–	–	–
	Infiltration, eosinophil, submucosa	–	–	–	–	–	–	–	–	–	1	–	–
Femur/Marrow	Increased cellularity	–	–	2	3	–	2	–	–	1	3	–	2
Ileum	Erosion/ulcer	–	–	–	1	–	–	–	–	1	–	–	–
	Infiltration, eosinophil, mucosa	–	–	–	–	–	–	–	–	–	1	–	–
Injection sites	Degeneration/regeneration, myofiber	2	3	–	–	–	–	2	3	–	–	–	–
	Hemorrhage	3	3	3	3	–	–	2	3	3	2	1	2
	Infiltration, mixed cell	2	2	–	–	–	–	–	–	–	–	–	–
	Infiltration, mononuclear cell	–	1	–	–	–	–	1	–	–	–	–	–
	Inflammation, chronic active	–	–	2	–	–	1	–	–	2	1	–	1
	Inflammation, granulomatous	–	–	1	3	–	1	–	–	1	2	–	1
	Mineralization	–	1	1	–	–	–	–	–	–	1	–	–
	Necrosis, myofiber	–	–	3	3	–	–	–	–	2	3	–	2
Jejunum	Intussusception	–	–	–	–	–	1	–	–	–	–	–	–
Kidney	Arteritis	–	–	–	1	–	–	–	–	–	–	–	–
	Dilation/basophilia, tubules	–	–	3	3	–	–	–	–	–	2	–	1
	Hydronephrosis	–	–	1	1	–	–	–	–	–	1	–	–
	Infiltration, mononuclear cell, interstitial/perivascular	1	1	2	3	–	1	1	1	2	2	–	–
	Necrosis, papilla	–	–	2	1	–	–	–	–	1	2	–	–
	Pigmentation, glomerulus	–	–	–	2	–	–	–	1	–	–	–	–
Liver with gallbladder	Infiltration, eosinophil, gallbladder	–	–	–	–	–	–	–	–	1	–	–	–
	Infiltration, mixed cell, limiting plate	–	–	2	3	–	2	–	–	2	3	–	1
	Infiltration, mononuclear cell, interstitial/periportal	2	2	1	–	–	–	1	3	1	–	–	–
	Pigmented histiocytes	–	–	–	1	–	–	–	–	–	–	–	–
Lung with bronchi	Alveolar macrophage aggregation	–	1	2	2	–	–	1	–	2	2	–	–
	Hemorrhage	–	–	1	1	–	1	–	–	–	–	–	–
Lymph node, mandibular	Pigment	1	–	–	–	–	–	–	1	1	–	–	–
Lymph node, mesenteric	Infiltration, neutrophil	–	–	1	2	–	–	–	–	2	1	–	–
	Pigment	1	3	1	1	–	–	–	1	–	–	–	–
Pituitary gland	Cyst(s)	–	–	1	–	–	–	–	–	–	–	–	–
Rectum	Infiltration, eosinophil	–	–	–	–	–	–	–	–	1	–	–	–
Salivary glands	Infiltration, mononuclear cell	1	–	–	–	–	–	–	1	–	–	–	–
Seminal vesicles	Infiltration, mononuclear cell	–	–	1	–	–	–	–	–	–	–	–	–
Skin	Erosion	–	1	–	–	–	–	–	1	–	–	–	–
	Erosion/Ulcer	–	–	2	3	–	–	–	–	–	2	–	–
	Infiltration, mixed cell	–	1	–	–	–	–	–	–	–	–	–	–
Sternum/Marrow	Increased cellularity	–	–	–	–	–	2	–	–	2	3	–	2
Stomach	Atrophy, mucosa	–	–	1	–	–	–	–	–	–	–	–	–
	Edema, submucosa	–	–	–	2	–	–	–	–	–	2	–	–
	Erosion/ulcer	–	–	–	1	–	–	–	–	1	2	–	–
	Infiltration, eosinophil, submucosa	–	–	–	2	–	–	–	–	1	1	–	–
	Infiltration, neutrophil, submucosa	–	–	–	1	–	–	–	–	–	1	–	–
	Ulceration	–	–	–	1	–	–	–	–	–	–	–	–
Testes	Infiltration, mixed cell, interstitial	–	–	2	2	–	–	–	–	–	–	–	–
	Degeneration, tubular	–	–	–	–	1	–	–	–	–	–	–	–
Thymus	Atrophy	–	–	1	2	–	2	–	–	–	1	–	1
Tongue	Ulceration	–	1	–	–	–	–	–	–	1	–	–	–

##### 3.7.3.1 Liver

**Fig. 2:**
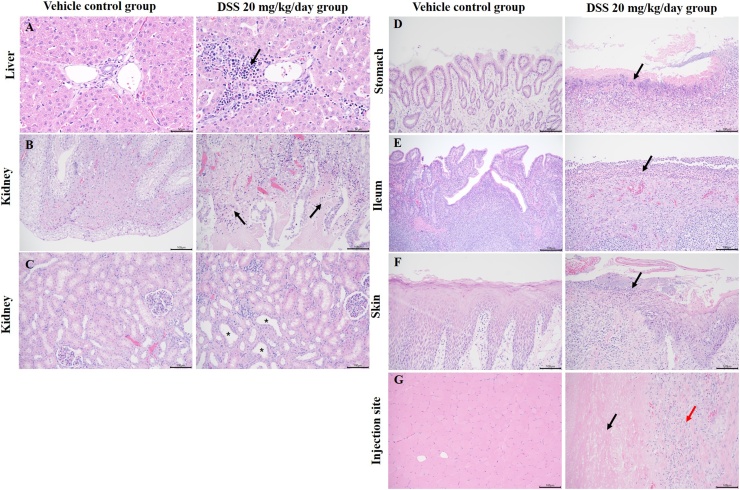
Representative images of histopathological findings in the liver, kidney, stomach, ileum, skin, and injection sites. (A) Inflammatory cell infiltration in the portal triad in the liver (right, black arrow)(x400) compared with that in the control vehicle group (left) (×400). The scale bar are 50 μm. (B) Necrosis at papillary tip in the kidney (right, black arrow) (×200) compared with that in the control vehicle group (left) (×200). The scale bar are 100 μm. (C) Tubular dilation and basophilia in the kidney (right, asterisk) (×200) compared with that in the control vehicle group (left) (×200). The scale bar are 100 μm. (D) Erosion/ulcers of the stomach (right, black arrow) (×200) compared with that in the control vehicle group (left) (×200). The scale bar are 100 μm. (E) Erosion/ulcers of the ileum (right, black arrow) (×200) compared with that in the control vehicle group (left) (×200). The scale bar are 100 μm. (F) Erosion/ulcers of the skin (right, black arrow) (×200) around the mouth compared with that in the control vehicle group (left) (×200). The scale bar are 100 μm. (G) Myofiber necrosis (black arrow) and granulomatous inflammation (red arrow) in injection site (right) (×200) compared with that in the control vehicle group (left) (×200). The scale bar are 100 μm. (Hematoxylin and eosin staining) (For interpretation of the references to colour in this figure legend, the reader is referred to the web version of this article).

Mixed cells were minimally to moderately infiltrated into the hepatic lobular margin in the middle-dose (2/3 males and 2/3 females) and high-dose (all males and females) groups receiving 10 and 20 mg/kg/day, respectively. Slight eosinophil filtration was observed in the gallbladder of one female in the middle-dose group receiving 10 mg/kg/day. Mixed cell infiltration was characterized by the invasion of numerous eosinophils and few mononuclear cells in the interstitial layer of the hepatic lobules and the hepatic portal vein (Fig. 2A). These changes were not observed in the recovery group.

##### 3.7.3.2 Kidney

Minimal-to-slight renal papillary necrosis was observed at the tip of the renal papillary in the middle-dose (2/3 males and 1/3 females) and high-dose (1/3 male and 2/3 female) groups receiving 10 and 20 mg/kg/day, respectively (Fig. 2B). In addition, minimal-to-slight tubular dilation and basophilia was observed in the middle-dose (3/3 males) and high-dose (3/3 males and 2/3 females) groups receiving 10 and 20 mg/kg/day, respectively (Fig. 2C).

##### 3.7.3.3 Stomach

Minimal-to-slight erosions/ulcers were noted in the middle-dose (1/3 female) and high-dose (1/3 male and 2/3 female) groups receiving 10 and 20 mg/kg/day, respectively (Fig. 2D). Marked submucosal edema in the stomach was observed in two animals of both sexes each at the high dose. Marked eosinophil and neutrophil infiltration was observed in the submucosa of males in the high-dose group (2/3 and 1/3, respectively) and of females in the middle dose (1/3 and 1/3, respectively) and high-dose group (2/3 and 1/3, respectively) receiving 10 and 20 mg/kg/day, respectively.

##### 3.7.3.4 Intestine (cecum, colon, and ileum)

Erosions/ulcers (minimal to slight) were observed in the cecum, colon, or ileum (Fig. 2E) of two males in the high-dose group and in all females in the middle-dose and high-dose groups receiving 10 and 20 mg/kg/day, respectively. Eosinophil infiltration (minimal to slight) was observed in all animals in the middle-dose group receiving 10 mg/kg/day.

##### 3.7.3.5 Mesenteric lymph node

Histological findings in the mesenteric lymph node showed minimal-to-moderate neutrophilic infiltration in the middle-dose (1/3 male and 2/3 female) and high-dose (2/3 males and 1/3 females) groups receiving 10 and 20 mg/kg/day, respectively.

##### 3.7.3.6 Sternum/marrow

There were minimal-to-slight increases in the cellularity in the sternum/marrow in the middle-dose (2/3 females) and high-dose (2/3 males and 3/3 females) groups receiving 10 and 20 mg/kg/day, respectively.

##### 3.7.3.7 Thymus

Slight-to-marked thymus atrophy was noted in the thoracic cavity in the middle-dose (1/3 male) and high-dose (2/3 males and 1/3 females) groups receiving 10 and 20 mg/kg/day, respectively.

##### 3.7.3.8 Skin

A moderate number of erosions/ulcers were found in the ear, lower forelimbs/hindlimbs, or around the mouth (Fig. 2F) in the middle-dose (2/3 males) and high-dose (3/3 males and 2/3 females) groups receiving 10 and 20 mg/kg/day, respectively. Slight-to-marked granulomatous inflammation and minimal-to-moderate myonecrosis were observed at the site of intramuscular injection (Fig. 2G) in the middle-dose (1/3 and 3/3 males as well as 1/3 and 2/3 females, respectively) and high-dose (3/3 and 3/3 males as well as 2/3 and 3/3 females, respectively) groups receiving 10 and 20 mg/kg/day, respectively. In addition, moderate-to-marked chronic active inflammation was noted at the injection site in the middle-dose (2/3 males and 2/3 females) and high-dose (1/3 female) groups receiving 10 and 20 mg/kg/day, respectively. These alterations were also retained during the recovery period.

### 3.8 Toxicokinetics

**Fig. 3:**
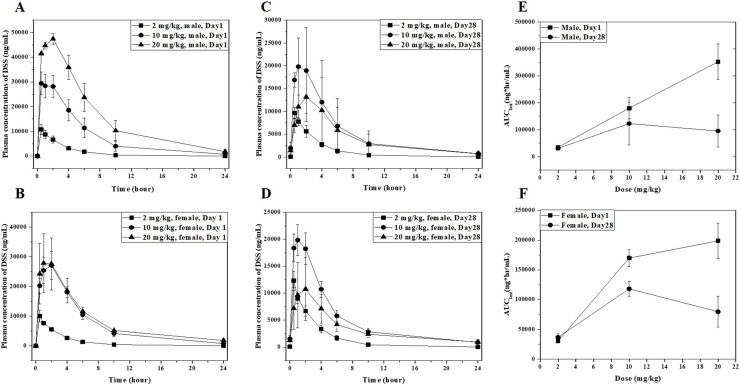
Mean plasma concentration of diclofenac sodium salt (DSS) in minipigs. Graph show the concentration of DSS in plasma over time in 3 males (A) and females (B) for each group on the first and last days of DSS administration. In A-D, the square represents the low-dose group (2 mg/kg/day), the circle represents the middle-dose group (10 mg/kg/day), and the triangle represents the high-dose group (20 mg/kg/day). The graph means the change in AUC_last_ by dose according to the administration day in males (E) and females (F). In E-F, the AUC_last_ on Day 1 after DSS administration was indicated by a square, and the AUC last on Day 28 was indicated by a circle.

The mean value and standard deviations of plasma-levels of all minipigs receiving 2, 10, or 20 mg/kg/day on Day 1 and Day 28 are given in Fig. 3. Table 8 summarized the mean and standard deviation of the toxicokinetic parameters following the intramuscular administration of diclofenac sodium salt (DSS) at dose of 2, 10 and 20 mg/kg to minipigs for four weeks. Systemic exposure (AUC_last_) was proportional to the dose by increasing about 1:5.2:10.1 in males (Fig. 3A) and 1:5.6:6.5 in females (Fig. 3B) as the doses increased to a ratio of 1:5:10 on Day1. There was a slightly lower rate of increase in the female high dose group receiving 20 mg/kg/day. The average value of T_max_ on Day 1 was 0.5−2 hours after administration. C_max_ was measured in the ratio of 1:2.8:4.4 in males and 1:2.7:2.9 in females as dose was increased on Day 1, which was similar to the trend of systemic exposure. The mean half-life (t_1/2_) was 3.06−5 hours in male and 3.76–7.35 hours in females, and increased with increasing dose. The systemic clearance (CL) was ranged 57.43–58.97 mL/hr/kg in females and 59.15–102.1 mL/hr/kg in females and the value was not changed with increasing dose.

**Table 8:** Toxicokinectic parameters of diclofenac in Minipig plasma.

	Male						Female					
	Day 1			Day28			Day 1			Day28		
Dose(mg/kg)	2	10	20	2	10	20	2	10	20	2	10	20
**K_el_** **(1/hr)**	0.227 ± 0.007	0.164 ± 0.058	0.141 ± 0.024	0.203 ± 0.011	0.115 ± 0.015	0.106 ± 0.015	0.194 ± 0.051	0.160 ± 0.052	0.097 ± 0.020	0.211 ± 0.015	0.098 ± 0.011	0.084 ± 0.020
**T_1/2_** **(hr)**	3.058 ± 0.101	4.684 ± 2.014	5.000 ± 0.789	3.419 ± 0.175	6.106 ± 0.833	6.653 ± 1.001	3.757 ± 1.099	4.736 ± 1.889	7.353 ± 1.504	3.295 ± 0.247	7.123 ± 0.751	8.590 ± 2.263
**T_max_** **(hr)**	0.500 ± 0.000	1.500 ± 0.866	2.000 ± 0.000	0.500 ± 0.000	1.167 ± 0.764	1.667 ± 0.577	0.500 ± 0.000	2.000 ± 0.000	1.333 ± 0.577	0.500 ± 0.000	1.000 ± 0.000	2.000 ± 0.000
**C_max_** **(ng/mL)**	10755.097 ± 2062.735	29891.629 ± 4724.753	47441.782 ± 2113.588	9678.806 ± 1154.921	20871.192 ± 7807.152	13446.726 ± 5344.507	10033.805 ± 2017.043	27082.993 ± 4649.740	29191.743 ± 8923.797	12315.545 ± 1761.022	19857.107 ± 2860.857	10782.109 ± 5844.791
**AUC_last_** (hr*ng/mL)	34902.447 ± 6779.075	180233.109 ± 40267.296	352576.276 ± 66186.682	30897.891 ± 4717.654	122880.431 ± 79824.662	95298.597 ± 59079.874	30512.692 ± 3609.608	169883.666 ± 14215.596	198826.227 ± 29938.352	36721.360 ± 6131.010	118162.481 ± 12691.116	79734.722 ± 25635.397
**AUC_inf_** (hr*ng/mL)	35002.752 ± 6787.384	186453.562 ± 42021.321	365702.717 ± 72165.958	31049.145 ± 4706.088	129220.469 ± 83971.292	101755.118 ± 62345.727	30824.961 ± 3853.879	176281.358 ± 19867.906	217638.338 ± 21857.039	36883.928 ± 6227.965	127027.389 ± 11911.891	92265.639 ± 32768.916
**CL** **(mL/hr/kg)**	58.968 ± 12.863	57.427 ± 13.157	57.956 ± 9.837	65.703 ± 9.593	105.240 ± 57.683	263.711 ± 134.917	66.209 ± 8.407	59.154 ± 5.201	102.097 ± 15.059	55.523 ± 9.553	85.288 ± 9.216	273.817 ± 107.370
**V_ss_** **(mL/kg)**	261.334 ± 66.028	382.328 ± 161.765	416.148 ± 90.177	325.519 ± 61.708	926.815 ± 485.001	2581.459 ± 1372.105	352.811 ± 77.827	399.724 ± 142.236	1104.761 ± 380.996	262.441 ± 35.838	883.029 ± 158.973	3243.983 ± 821.217

**K_el_**, elimination rate constant; **T_1/2_**, elimination half-life; **T_max_**, time to maximum plasma concentration(C**_max_**); **C_max_**, maximum plasma concentration; **AUC,** Area under the concentration-time curve; **CL,** clearance; **V_ss_**, volume of distribution at steady state.

After repeated administration for 4 weeks, systemic exposure (AUC_last_) to diclofenac sodium salt was 89–120 % at the low dose group receiving 2 mg/kg/day of both sexes compared to that on Day 1, and there was no difference according to diclofenac sodium salt repeated administration (Fig. 3E-F). However, systemic exposure (AUC_last_) at 10 mg/kg and 20 mg/kg dose groups showed a tendency to decrease to 68–70 % and 27–40 %, respectively (Fig. 3E-F).

Systemic exposure (AUC_last_) of diclofenac sodium salts in females was indicated in 56–94 % on Day 1 and 84–119 % on Day 28 compared to males. A gender difference was not observed (< 2 fold) in systemic exposure (AUC_last_) of whole groups except for 20 mg/kg dose group on Day 1 (Fig. 3E-F).

## 4 Discussion

The toxicity of diclofenac sodium salt (DSS) has been assessed in various animal studies [2,11,12,14]. Gastrointestinal (GI)-related bleeding or anorexia were observed in acute toxicity study, and LD50 was between 95–1300 in mice, 53–1500 mg/kg in rats, 125–300 mg/kg in rabbits, 1110–1250 mg/kg in guinea pigs, 59–800 mg/kg in dogs and 3200 mg/kg in monkeys [14,20]. GI, hepatic, and renal dysfunctions were observed in chronic toxicity studies through oral, dermal or subcutaneous administration from about 1 week to 1 year, and No-observed-adverse-effect-level (NOAEL) was 2.5 mg/kg in rat administered DSS orally for 91 days, 7 mg/kg in rat administered DSS intravenously for 4 weeks and 3 mg/kg in monkey administered DSS intravenously for 4 weeks [20,21]. According to the results of dermal administration toxicity study for 9 weeks or 6 months in minipigs, not only the toxicity symptoms mentioned above, but also skin reactions not observed in other animal species were observed in pigs. However, sporadic erythema or dermatitis at the injection site was observed, and no other skin changes were observed [14].

Our study was conducted to evaluate the toxicity of DSS after 4 weeks of repeated intramuscular administration once daily in minipigs and to assess recovery for 2 weeks. A total of 32 minipigs were used in this study, and they were categorized into four dosing groups: 0, 2, 10, and 20 mg/kg/day. Each group comprised three males and females each, whereas the 0 mg/kg/day (vehicle control group) and 20 mg/kg/day groups, which were assigned as recovery groups, had additional two males and females. No deaths due to DSS administration were noted during the experimental period; however, significant changes in clinical signs; and hematological, clinical chemistry, and urinalysis parameters; organ weight; and macro/microscopic examination results were observed.

DSS-related renal parameters changes such as renal papillary necrosis, tubular dilation, and basophilia were observed at 10 mg/kg/day in males and 20 mg/kg/day in both sexes. These parameters included the colored urine observed in clinical analysis, reductions in total protein and albumin in clinical chemistry analysis, absolute or relative kidney weight increases in organ weight analysis, and paleness of the kidney in macroscopic examination. These changes were considered the adverse effects of DSS and showed a tendency to be resolved during the recovery period.

In the liver, mixed cell infiltration of the hepatic lobular margin was observed in both sexes in the 10 and 20 mg/kg/day groups, and eosinophil infiltration of the gallbladder was observed in females in the 10 mg/kg/day group. In this regard, increases in the number of leukocytes, monocytes, and eosinophils were observed in the hematological examination, as well as an increase in the absolute or relative weight of the liver. All these change were likely related to DSS administration, and there was a tendency for these to be resolved after the recovery period.

Histopathological examination of the GI tract (stomach, ileum, cecum, or colon) revealed erosions/ulcers in the 10 (female) and 20 mg/kg/day (both sexes) groups. With regard to these changes in the GI tract, vomiting, a reduction in the erythrocyte index (erythrocyte count, hemoglobin concentration, and hematocrit), and an increase in mean RBC volume and platelet count were observed. These findings were also considered to be adverse effects related to DSS and showed a tendency to be resolved after the recovery period.

In addition, although only one male in the high-dose group receiving 20 mg/kg/day showed an ulcer at the gastric retinal adhesion site, this finding was considered to be the effect of DSS because ulcer induction was consistently observed other animals administered DSS. Eosinophils or neutrophils infiltrated the GI tract and mesenteric lymph nodes in both sexes in the 10 and 20 mg/kg/day groups. These were considered to be associated with an increase in WBC count, neutrophil count and ratio, and eosinophil count observed in the hematological examination as well as with the red discoloration and enlargement of the mesenteric lymph nodes observed in the macroscopic analysis. These observations were induced by the administration of DSS and accompanied recovery.

In the bone marrow (sternum and femur), increased cellularity was observed in both sexes in the 10 and 20 mg/kg/day groups, as determined by the hematological examination. This was associated with an increase in the number and ratio of reticulocytes. These changes were assumed to be secondary hematopoietic changes in the RBC inflammation owing to the administration of DSS and were not adverse effects [22].

In the thymus, atrophy was observed in the thoracic cavity in males in the 10 mg/kg/day group and in males and females in the 20 mg/kg/day group, and this persisted throughout the recovery period. These findings were correlated with a decreased thymus size, as determined by macroscopic analysis. This was considered a secondary change caused by stress rather than by DSS administration and was not considered an adverse effect of DSS [23].

At the injection site, granulomatous inflammation, muscle fiber necrosis, and chronic active inflammation, which is observed in histopathology, were observed in both male and females in the middle- and high-dose groups receiving 10 and 20 mg/kg/day, respectively. These were considered as toxicities of DSS associated with swelling of the neck observed via the clinical observation and discoloration observed via the macroscopic examination at the administration site in males and females in the 10 and 20 mg/kg/day groups. Similar changes were observed even after the recovery period.

In the skin (ear, limb, or mouth), erosions/ulcers were observed in males and females in the 10 and 20 mg/kg/day groups, and these were associated with the swelling of the skin, abscesses, ulcers, and swelling of the palate. Therefore, these symptoms were considered toxicities of DSS treatment [7,24]. Some animals showed evidence of limping; however, there were no changes in related blood clinical chemistry, and thus, this was considered to be a secondary change caused by skin damage, such as swelling, abscesses, and ulcers of the forelimb/hindlimb. In addition, loss of teeth was considered a secondary change caused by swelling of the palate and ulceration of the lips. Swelling of the palate was observed to a similar degree during the recovery period. Other symptoms such as scratches, scars, and scabs were considered unrelated to DSS administration. Rather, these were caused by fights between animals when two animals were housed in one cage. Subsequently, such symptoms were not observed or showed a tendency to recover when the animals were reared alone. In reports that DSS administered dermally for 30 days to 6 months in minipigs receiving 0, 3, 10 and 30/45 mg/kg, skin reactions such as sporadic erythema or dermatitis at only application sites were observed [14]. However, swelling, abscesses, and ulcers were observed not only at the site of administration but also at the sites such as the ear, limb or mouth. In toxicokinetics results, the systemic exposure (AUC_last_) of the DSS administration intramuscularly on Day1 is about 380 times higher than dermal administration based on 10 mg/kg/day [14]. The AUC_last_ following intramuscular administration at a dose 2.5 mg/kg/day similar to that of a low dose receiving 2 mg/kg in this study [25,26].

The systemic exposure (AUC_last_) of the DSS on Day1 was similar to the increase rate of the dose in both sexes’ animals or slightly lower in the 20 mg/kg dose group of female animals. Systemic exposure of the diclofenac sodium salt after four weeks of repeated administration showed a decreasing trend in the male and female animals with a dose of 10 mg/kg or more compared to Day 1. Systemic exposure of the diclofenac sodium salt was not significantly different between male and female animals in the other dose groups except for the 20 mg/kg dose group (56 %) on Day 1. It has been reported that the DSS was well tolerated after the IM injection of the DSS at a dose of 75 mg in humans (Leuratti et al., 2019). If the human effective dose (HED) used in the above literature converts to animal dose (minipig correction factor (Km), 35; human Km, 37), the animal dose is around 1.4 mg/kg. Also, the DSS was well tolerated in rats which are intravenously received the DSS at a dose of 7 mg/kg [21]. If the Km is applied to convert this dose, the effective mini pig dose is around 1.2 mg/kg. In this study, the DDS was well tolerated after IM injection of DDS at 2 mg/kg in mini pig, while the mini pig, which is received the DDS at dose of 10 and 20 mg/kg, exhibited toxicity. According to the EMEA report [20], DSS is intended for treatment in cattle and swine as an anti-inflammatory agent at doses of 2.5 mg/kg bw/day by intramuscular route for 1–3 days. As described above, no evidence of systemic toxicity was observed after IM injection of the DSS at 2 mg/kg for 28 days. However, the DDS treatment groups at 10 and 20 mg/kg showed systemic toxicity, thus, the increasing dose or extending treatment period of DDS should be cautioned in clinical condition.

In conclusion, intramuscular administration of DSS daily resulted in GI, renal, hepatic and skin toxicities as well as injection-site reactions in both males and females receiving 10 mg/kg/day or higher dose groups. These changes were observed systemically and considered to be adverse effects associated with DSS. The NOAEL of DSS in this 4-week repeat-dose toxicity study was considered to be 2 mg/kg/day in both sexes of minipigs.

## Declaration of Competing Interest

The authors declare that they have no known competing financial interests or personal relationships that could have appeared to influence the work reported in this paper.no conflict of interest.

## References

[1] El-Maddawy Kh, El-Ashmawy Ibrahim (2013). Hepato-renal and hematological effects of diclofenac sodium in rats. Glob. J. Pharmacol..

[2] Ahmad I., Qureshi T.A., Sadique U., Khan S.A., Ahmed S., Rehman Z.U., Baharar S., Mushtaq M. (2013). Hematological effects of diclofenac sodium in goat. J. Anim. Plant Sci..

[3] Gomaa S. (2017). Immunomodulatory and hematological effects induced by diclofenac, ibuprofen or paracetamol toxicity in Swiss albino mice. Eur. J. Biol. Res..

[4] Aycan I.O., Elpek O., Akkaya B., Kirac E., Tuzcu H., Kaya S., Coskunfirat N., Aslan M. (2018). Diclofenac induced gastrointestinal and renal toxicity is alleviated by thymoquinone treatment. Food Chem. Toxicol..

[5] Gupta A., Kumar R., Ganguly R., Singh A.K., Rana H.K., Pandey A.K. (2020). Antioxidant, anti-inflammatory and hepatoprotective activities of Terminalia bellirica and its bioactive component ellagic acid against diclofenac induced oxidative stress and hepatotoxicity. Toxicol. Rep..

[6] Adeyemi W.J., Omoniyi J.A., Olayiwola A., Ibrahim M., Ogunyemi O., Olayaki L.A. (2019). Elevated reproductive toxicity effects of diclofenac after withdrawal: investigation of the therapeutic role of melatonin. Toxicol. Rep..

[7] O’brien W.M. (1986). Adverse reactions to nonsteroidal anti-inflammatory drugs; diclofenac compared with other nonsteroidal anti-inflammatory drugs. Am. J. Med..

[8] Boelasterli U.A. (2003). Diclofenac-induced liver injury: a paradigm of idiosyncratic drug toxicity. Toxicol. Appl. Pharmacol..

[9] Gor A.P., Saksena M. (2011). Adverse drug reactions of nonsteroidal anti-inflammatory drugs in orthopedic patients. J. Pharmacol. Pharmacother..

[10] Adeyemi W.J., Olayaki L.A. (2018). Diclofenac-induced hepatotoxicity: low dose of omega-3 fatty acids have more protective effects. Toxicol. Rep..

[11] Thanagari B.S., Fefar D.T., Prajapati K.S., Jivani B.M., Thakor K.B., Patel J.H., Ghodasara D.J., Joshi B.P., Undhad V.V. (2012). Haemato-biochemical alterations induced by diclofenac sodium toxicity in Swiss albino mice. Vet. World.

[12] Khazaeinia T., Jamali F. (2003). A comparison of gastrointestinal permeability induced by diclofenac-phospholipid complex with diclofenac acid and its sodium salt. J. Pharm. Sci..

[13] Vyas A., Purohit A., Ram A. (2019). Assessment of dose-dependent reproductive toxicity of diclofenac sodium in male rats. Drug Chem. Toxicol..

[14] NDA 21-005 (2000). Pharmacology review(s). https://www.accessdata.fda.gov/drugsatfda_docs/nda/2000/21005_Solaraze_pharmr_P2.pdf.

[15] Ramzan M., Ashraf M., Hashmi H.A., Iqbal A., Anjum A.A. (2015). Evaluation of diclofenac sodium toxicity at different concentrations in relation to time using broiler chicken model. J. Anim. Plant Sci..

[16] Rahal A., Kumar A., Ahmad A.H., Malik J.K. (2008). Pharmacokinetics of diclofenac and its interaction with enrofloxacin in sheep. Res. Vet. Sci..

[17] Yuan J., Ma J., Cen N., Zhou A., Tao H. (2017). A pharmacokinetic study of diclofenac sodium in rats. Biomed. Rep..

[18] Gardner J.D., Calkins J.B., Garrison G.E. (2014). ECG diagnosis: the effect of ionized serum calcium levels on electrocardiogram. Clin. Med..

[19] Ruppert S., Vormberge T., Bernd-Wolfgang, Hoffmann M. (2016). ECG telemetry in conscious guinea pigs. J. Pharmacol. Toxicol. Methods.

[20] EMEA (2003). Committee for verterinary medicinal products diclofenac summary report. The European Agency for the Evaluation of Midecinal Products (The European Agency for the Evaluation of Midecinal Products), UK, September 2003.

[21] NDA 22-396 (2014). Pharmacology review(s). https://www.accessdata.fda.gov/drugsatfda_docs/nda/2014/022396Orig1s000PharmR.pdf.

[22] Richard W.L., Richard B., Eric D., Armin G., Lang B., Francis C. (2002). Recognition of adverse and nonadverse effects in toxicity studies. Toxicol. Pathol..

[23] Nancy E.E., Paul W.S., Keith L.B., Brad B., Dianne M.C., George L.F., Thomas J.R., Teresa S. (2013). Interpreting stress responses during routine toxicity studies; A review of the biology, impact, and assessment. Toxicol. Pathol..

[24] Brogden R.N., Heel R.C., Pakes G.E., Speight T.M., Avery G.S. (1980). Diclofenac sodium: a review of its pharmacological properties and therapeutic use in rheumatic diseases and pain of varying origin. Drugs.

[25] Yang H.F., Li Y.J., Li Y.Y., Huang C., Huang L.X., Bu S.J. (2019). Pharmacokinetics of diclofenac sodium injection in swine. Pol. J. Vet. Sci..

[26] Zorica P., Milena P., Milanka J. (2006). Pharmacokinetics of diclofenac in pigs after intramuscular administration of a single dose. Acta Vet. (Beograd)..

